# PES Pathogens in Severe Community-Acquired Pneumonia

**DOI:** 10.3390/microorganisms7020049

**Published:** 2019-02-12

**Authors:** Catia Cillóniz, Cristina Dominedò, Antonello Nicolini, Antoni Torres

**Affiliations:** 1 Department of Pneumology, Hospital Clinic of Barcelona—Institut d’Investigacions Biomèdiques August Pi i Sunyer (IDIBAPS), University of Barcelona (UB)—SGR 911—Ciber de Enfermedades Respiratorias (Ciberes), 08036 Barcelona, Spain; catiacilloniz@yahoo.com; 2 Department of Anesthesiology and Intensive Care Medicine, Fondazione Policlinico Universitario A. Gemelli, Università Cattolica del Sacro Cuore, 00168 Rome, Italy; c.dominedo1@alice.it; 3 Respiratory Diseases Unit, Hospital of Sestri Levante, 16039 Sestri Levante, Italy; antonellonicolini@gmail.com

**Keywords:** pneumonia, PES pathogens, severe pneumonia, community-acquired pneumonia

## Abstract

Worldwide, there is growing concern about the burden of pneumonia. Severe community-acquired pneumonia (CAP) is frequently complicated by pulmonary and extra-pulmonary complications, including sepsis, septic shock, acute respiratory distress syndrome, and acute cardiac events, resulting in significantly increased intensive care admission rates and mortality rates. *Streptococcus pneumoniae* (Pneumococcus) remains the most common causative pathogen in CAP. However, several bacteria and respiratory viruses are responsible, and approximately 6% of cases are due to the so-called PES (*Pseudomonas aeruginosa*, extended-spectrum β-lactamase *Enterobacteriaceae*, and methicillin-resistant *Staphylococcus aureus*) pathogens. Of these, *P. aeruginosa* and methicillin-resistant *Staphylococcus aureus* are the most frequently reported and require different antibiotic therapy to that for typical CAP. It is therefore important to recognize the risk factors for these pathogens to improve the outcomes in patients with CAP.

## 1. Severe Community-Acquired Pneumonia: What Is the Current Definition?

Currently, there is no consensus for the definition of severe community-acquired pneumonia (CAP) largely because it includes such a heterogeneous patient group. The most widely accepted definition is based on the 2007 Infectious Diseases Society of America/American Thoracic Society consensus (ATS/IDSA) guidelines for the management of CAP in adults [1]. According to the ATS/IDSA guidelines, severe CAP is defined by the presence of two major criteria: the need for invasive mechanical ventilation (IMV) due to severe acute respiratory failure and/or the presence of septic shock (Table 1). Several minor criteria requiring high intensity monitoring and treatment have also been proposed [1].

## 2. Admission to Intensive Care Units: What Is the Real Impact?

During recent decades, the numbers of patients with pneumonia requiring management in intensive care has grown globally. The aging population [2] and the increasing number of immunocompromized patients [3,4,5] (e.g., due to solid organ and hematopoietic stem cell transplantation, human immunodeficiency virus, or biological or immunosuppressive therapies) probably explains much of this change.

The percentage of admissions to intensive care units (ICUs) that are attributable to elderly patients ranges from 9% to 19% in Europe [6,7,8,9,10,11] and from 20% to 30% in America [12]. A recent French study of ICU admission trends among elderly patients (≥75 years) between 2006 and 2015 indicated that 3% of all hospitalizations (3,856,785 cases) were for an acute respiratory infection (ARI) (98,381 cases) and that 15% of these required ICU admission (15,267 cases). The authors noted that the annual number of ICU hospitalizations increased steadily from 740 to 2034 during the study, with a 2.7-fold increase in ICU admissions for respiratory infection (*p* = 0.002). There was an overall increase in the number of ICU admissions for all age groups but with the greatest increases in ICU resources seen for patients aged 85–89 years (3.3 fold) and ≥90 years (5.8 fold). Interestingly, the increased ICU admission rate was not associated with significant changes in the ICU mortality rate for patients with ARI, with rates of 19.7% ± 3.0%, 24.0% ± 3.6%, and 25.0% ± 4.0% for those aged 75–79, 80–84, and 85–89 years, respectively. Indeed, there was a significant drop in ICU mortality from 40.9% in 2006 to 22.3% in 2015 (*p* = 0.03) for patients aged ≥90 years [13]. Finally, they reported that hospitalizations for CAP and acute exacerbations of chronic obstructive pulmonary disease increased significantly for all age groups during the study period.

## 3. Outcomes of Patients with Severe Cap: What Are the Main Determinants?

Despite improvements in management, severe CAP is associated with significant mortality. It is known that patients with respiratory failure and IMV, sepsis, septic shock, and decompensated comorbidities are at greater risk of death [14,15,16,17,18]. In the study by Jain et al. [19] in which 2320 cases of pneumonia were analyzed, approximately 21% of patients with CAP required ICU admission and 6% required IMV [19]. More recently, a Spanish study [14] of 154 severe CAP cases found a higher 30-day mortality rate (33%) in patients receiving IMV compared with non-intubated patients (18%). Patients receiving IMV did not present higher severity scores at hospital admission according to APACHE-II, PSI, or CURB-65 scores, but the use of IMV independently predicted 30-day mortality. The authors concluded that, based on these results, the PSI, CURB-65, and APACHE-II scores were less suitable than IMV for reliably identifying patients with severe CAP at higher risk of mortality. IMV, septic shock, worsening hypoxemia, and increased serum potassium were independently associated with increased mortality.

**Figure 1:**
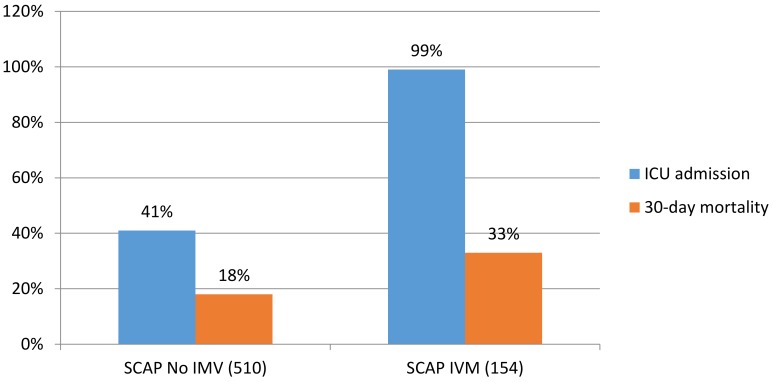
The outcomes in patients with severe community-acquired pneumonia (SCAP) with non-invasive ventilation (NIV) and invasive mechanical ventilation (IMV). Intensive care unit (ICU). X–axis showed the percentage and y-axis showed study population. Adapted from Reference [21].

Interestingly, a recently published systemic review and meta-analysis [20] that included nine studies on the use of noninvasive ventilation (NIV) in acute hypoxemic respiratory failure showed a protective effect for intubation and mortality with the use of NIV in patients with acute pulmonary edema, CAP, or immunosuppression (Figure 1).

Cillóniz et al. [15] investigated acute respiratory distress syndrome (ARDS) in mechanically ventilated patients with severe CAP. Of the 5334 participants, 930 (17%) were admitted to the ICU, 462 (52%) were not ventilated, 137 (15%) received NIV, and 295 (33%) received IMV; 125 cases (29%) met the Berlin ARDS criteria [22]. ARDS affected 2% of all patients hospitalized with CAP and 13% of patients admitted to the ICU. According to the severity of ARDS, the 30-day mortality rates were 32%, 33%, and 60% for patients with mild, moderate, and severe ARDS, respectively.

Sepsis is another important complication of severe CAP [17]. The global incidence of hospital-treated sepsis has been estimated at 31.5 million for sepsis and 19.5 million for severe sepsis, with a potential 5.3 million annual deaths in high-income countries [23]. Sepsis is associated with prolonged ICU stays and high mortality rates (20%–30%), with those rates increasing when shock is present to approximately 45% [21].

In 2016, a Spanish study investigated the predictors of severe sepsis among 4070 hospitalized patients with CAP. Of these, 37% presented with severe sepsis (1529 patients), of which 67% were ≥65 years and 63% had PSI risk class IV–V. The 30-day mortality of septic patients (7%) was significantly higher than that in non-septic patients (3%; p 0.001). Predictors of severe sepsis were older age, alcohol abuse, renal disease, and chronic obstructive pulmonary disease, whereas prior antibiotic treatment was a protective factor [17].

Georges et al. [24] investigated the prognosis of patients admitted to the ICU with CAP after the implementation of new care strategies, including sepsis bundles derived from the Surviving Sepsis Campaign [25], an initial empirical antimicrobial regimen with a third-generation cephalosporin and levofloxacin, and the use of NIV following extubation. Comparing the pre-implementation period (1995–2000) with the implementation period (2005–2010), mortality decreased from 43.6% to 30.9% (*p* < 0.02). Consistent with these results, Gattarello et al. [26], in a matched case-control study (80 cases and 80 controls) comparing 2000–2002 and 2008–2013, there was a 15% decrease in mortality among patients with pneumococcal pneumonia admitted to ICU. Early antibiotic administration and combination antibiotic therapy were both independently associated with better outcomes.

## 4. Pathogens Beyond the Core Microorganisms of Cap: Should We Be Worried About Them?

The most frequent pathogens outside the core microorganisms of CAP are methicillin-resistant *Staphylococcus aureus* (MRSA), *Pseudomonas aeruginosa*, *Acinebacter baumannii*, and various *Enterobacteriaceae* [27,28]. Since antibiotic therapy for these pathogens is different from the usual empirical therapy for CAP, it is important to recognize their main risk factors to ensure early diagnosis and appropriate treatment.

In 2012, an international expert proposal for interim standard definitions for acquired resistance was published to allow data comparison and to improve comprehension of the real problem of antimicrobial resistance globally. Magiorakos et al. proposed the following definition: multidrug resistance (MDR, or resistance to at least one agent in ≥3 antibiotic groups), extensively drug resistance (XDR, or resistance to at least one agent in all but ≤2 antibiotic groups), and pan drug resistance (PDR, or resistance to all antibiotic groups) [29] (Table 2). In the same year, Aliberti et al. [30] analyzed 935 patients. Among them, 473 (51%) had at least 1 risk factor for MDR pathogen on admission. The authors proposed a score that included the following variables: chronic renal failure (5 points), prior hospitalization (4 points), nursing home residence (3 points), and other variables (0.5 points each for cerebrovascular disease, diabetes, chronic obstructive pulmonary disease, immunosuppression, home wound care, prior antimicrobial therapy, and home infusion therapy). The prevalence of resistant pathogens was 38% in patients with a score of ≥3 points, compared with 8% in patients with a score of ≤0.5. The overall prevalence of PES pathogens in this study was lower than in others. The thresholds 0–0.5 points and 3–12.5 points were associated with low and high risks of MDR, respectively.

In 2013 a study about MDR pathogens in two independent European cohorts of hospitalized patients with CAP was published. MDR pathogens were identified in 3.3% of patients in the Spanish cohort and in 7.6% of patients in the Italian cohort, with MRSA being the most common. In both cohorts, there was a significantly higher prevalence of MDR bacteria among patients in the ICU compared with patients treated on the ward [27].

In 2015, Prina et al. [31] proposed the PES score, based on the three most frequent MDR pathogens in CAP (e.g., *P. aeruginosa*, extended-spectrum β-lactamase-positive *Enterobacteriaceae*, and methicillin-resistant *S. aureus*). The following elements were included: 1 point each for age 40–65 years and male sex; 2 points each for age >65 years, previous antibiotic use, chronic respiratory disorder, and impaired consciousness; 3 points for chronic renal failure; and minus 1 point if fever was present initially. The thresholds ≤1 point, 2–4 points, and ≥ 5 points indicated low, medium, and high risks of MDR, respectively (Table 3).

Also in 2015, a score was developed by Falcone et al. [28] (the ARUC score) based on the following: 1 point for healthcare-associated pneumonia (HCAP) criteria (defined by the presence of at least one of hospitalization in the previous 3 months, dialysis, intravenous chemotherapy in the past 30 days, admission to an acute care hospital for at least 2 days, surgery in the past 90 days, or resided in a nursing home or long-term care facility); 0.5 points for bilateral pulmonary infiltrations and pleural effusion; and 1.5 points for a PaO2/FiO2 <300. Patients were then stratified as low (<0.5 points) or high (≥3 points) risk for MDR pathogens. The authors analyzed 300 patients with an etiologic diagnosis of CAP or HCAP, of which 99 (11%) presented MDR pathogens; only 12% of these required an ICU admission.

In 2016, Webb et al. [32] proposed the DRIP (drug resistance in pneumonia) score based on major and minor risk factors. Major risk factors (2 points) included prior antibiotics, residence in a long-term care facility, tube feeding, and prior infection with a drug-resistant pathogen (1 year) and minor risk factors (1 point) included hospitalization within the previous 60 days, chronic pulmonary disease, poor functional status, gastric acid suppression, wound care, and MRSA colonization (1 year). A threshold of ≥4 points identified patients at high risk of pneumonia due to a drug-resistant pathogen.

In 2017, Ishida et al. [33] evaluated the risk factors for antimicrobial-resistant pathogens in immunocompetent patients with pneumonia and validated the role of PES pathogens in this subgroup of patients. Among the 1559 patients with CAP, an etiological diagnosis was reached in 45% of patients and PES pathogens were identified in 7%. Patients with PES pathogens showed a trend toward initial treatment failure, readmission within 30 days, and prolonged hospital stays. Risk factors associated with infection by PES pathogens were female sex, admission within 90 days, poor performance status, and enteric feeding. The authors concluded that the concept of PES pathogens provided an appropriate description of drug-resistant pathogens associated with pneumonia in immunocompetent patients.

Recently, the European Antimicrobial Resistance Surveillance Network (EARS-Net) published its data regarding the burden of infections caused by antibiotic-resistant bacteria in countries of the EU and European Economic Area in 2015 [34]. The EARS-Net estimated that there were 671,689 infections with antibiotic-resistant bacteria, of which 64% (426,277) were health care associated. These infections accounted for an estimated 33,110 attributable deaths and 874,541 disability-adjusted life-years. Infants (aged <1 year) and the elderly (≥65 years) had higher burdens, as did Italy and Greece. Moreover 80% of the total disability-adjusted life-years per 100,000 were caused by infection with four pathogens: third-generation cephalosporin-resistant *Escherichia coli*, MRSA, third-generation cephalosporin-resistant *Klebsiella pneumoniae*, and carbapenem-resistant *Pseudomonas aeruginosa*.

### Risk Scores for Specific Pathogens (MRSA and P. aeruginosa)

In 2013, Shorr et al. [35] analyzed 5975 patients admitted with bacterial pneumonia. MRSA was identified in 14% of patients. The authors proposed a new score that included eight variables: recent hospitalization or ICU admission (2 points), age < 30 or > 79 years (1 point), prior IV antibiotic exposure (1 point), dementia (1 point), cerebrovascular disease (1 point), female with diabetes (1 point), or recent exposure to a nursing home/long term acute care facility/skilled nursing facility (1 point). The prevalence of MRSA was < 10% in low risk patients (0–1 points), approximately 22% in medium risk patients (2 to 5 points), and >30% in high risk patients (6 or more points).

In 2018, Restrepo et al. [36] published data of a multinational point prevalence study enrolling 3193 patients in 54 countries with confirmed diagnoses of CAP and who underwent microbiological testing at admission. The authors reported that the prevalence of *P. aeruginosa* was 4.2% and the prevalence of antibiotic-resistant *P. aeruginosa* was 2.0%. The authors identified the following risk factors for *P. aeruginosa* infection: prior *Pseudomonas* infection, tracheostomy, bronchiectasis, invasive respiratory and/or vasopressor support, and severe COPD. Conversely, risk factors for antibiotic-resistant *P. aeruginosa* infection were past medical history of a pseudomonas infection or tracheostomy. According to the recommendations provided by the authors, in the absence of specific risk factors, an empiric antibiotic therapy with a β-lactam plus a respiratory fluoroquinolone or a macrolide is effective against most pathogens responsible for CAP. Empiric antipseudomonal should be limited to patients with a past medical history of pseudomonas infection and/or chronic lung diseases, independently of disease severity.

In conclusion, the continuous increase of drug resistance among virulent pathogens represents a major challenge for clinicians and health care providers. Of course, the identification of risk factors is a key element for the management of pathogens beyond those typical in CAP. We believe that the concept of PES pathogens provides an accurate description of drug resistance in immunocompetent patients with CAP.

## 5. Empiric Antibiotic Therapy in Severe CAP Caused by PES Pathogens: Is There Something New?

A recent study by Marayuma et al. [37] proposed an antibiotic strategy for pneumonia based on the risk factors for PES pathogens, independent of the site of pneumonia acquisition. Risk factors for PES pathogens were antibiotic therapy in the past 180 days, poor functional status (Barthel Index <50 or performance status ≥3), hospitalization for >2 days in the past 90 days, occurrence of pneumonia ≥5 days after admission to an acute hospital, requirement for hemodialysis, and immunosuppression. The authors prospectively applied the therapeutic algorithm to a multicenter cohort of 1089 patients, of whom 656 had CAP, 238 had HCAP, 140 had hospital-acquired pneumonia, and 55 had ventilator-associated pneumonia. Patients with 0–1 risk factors for PES pathogens were treated with standard therapy (a β-lactam plus a macrolide), whereas patients with ≥2 risk factors for PES pathogens were treated with a appropriate therapy for hospital-acquired pneumonia (a two- or three-drug regimen combining an antipseudomonal β-lactam with a quinolone or aminoglycoside plus optional linezolid or vancomycin). Approximately 83% of patients were treated according to the proposed algorithm, and 4% received inappropriate therapy. The authors concluded that using an algorithm based on the risk factors for PES pathogens and disease severity rather than the site of pneumonia acquisition, simplified treatment, improved the accuracy of empiric therapy, and reduced mortality, avoiding the overuse of broad-spectrum antibiotics in some patients. Although this algorithm may be promising, it has only been validated in Japan. It will, therefore, be necessary to validate the algorithm in other countries, health care systems, and clinical settings.

Currently, the empiric antibiotic therapy for severe CAP remains based on international guidelines that recommend using a macrolide or a respiratory fluoroquinolone in combination with a β-lactam [1,38]. The coverage for PES pathogens should only be given if risk factors are present. Unfortunately, the superiority of a β-lactam plus a macrolide compared to a β-lactam plus a fluoroquinolone in the treatment of severe CAP remain unconfirmed. A recent meta-analysis [39] of patients with severe CAP showed that patients receiving a β-lactam plus a macrolide were discharged from the hospital about 3 days earlier than patients treated with a β-lactam and fluoroquinolone. The overall mortality also differed significantly between the groups, with rates of 19% for β-lactam plus macrolide therapy and 27% for β-lactam plus fluoroquinolone therapy. However, the length of ICU stay did not differ between the groups. More recently, a Spanish study [40] investigated the effect on mortality of a combined β-Lactam/macrolide therapy for CAP according to the etiology and the inflammatory status (measured by the levels of C-reactive protein). The study included 1,715 CAP patients with known etiology; the authors found that the combination of a β-lactam with a macrolide was associated with a lower mortality in patients with pneumococcal CAP and in patients with high systemic inflammatory response. However, two randomized controlled trials (RCTs) showed that the combination therapy with a β-lactam and a macrolide did not significantly reduce the mortality of non-ICU CAP patients [41,42].

**Figure 2:**
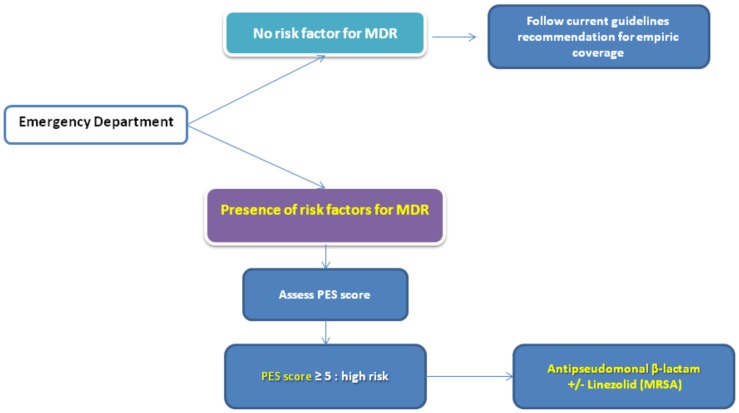
The management of severe community-acquired pneumonia.

We recommend following the current international guidelines for empiric therapy in cases of severe CAP [1] and to use the PES score [31] to identify patients at risk for PES pathogens. The use of empiric antibiotics that cover PES pathogens should be used in patients with a PES score ≥ 5 points) (Figure 2).

## 6. Are There Any New Antibiotics for PES Pathogens in Severe CAP?

The research and development of new antibiotics is scientifically and economically challenging, yet it remains an essential goal given current global needs and the spread of antimicrobial resistance. In the European Union alone, it has been calculated that approximately 25,000 deaths are caused by antibiotic-resistant microorganisms each year, with the global burden estimated to be 700,000 deaths per year [43]. The production of new antibiotics has declined in recent decades, however, and between 2010 and 2018, only eight new antibiotics were registered.

Ceftobiprole is a broad-spectrum cephalosporin that has activity against many pathogens that cause pneumonia, including gram-positive pathogens, such as penicillin-resistant *Streptococcus pneumoniae* (PRSP), MRSA, and various gram-negative pathogens (including *Pseudomonas* species). Ceftobiprole blocks the transpeptidase activity of the penicillin binding proteins in gram-positive and gram-negative pathogens. This causes the synthesis of peptidoglycan to decrease and the bacterium to die by osmosis or by autolysis. The bactericidal activity of ceftobiprole is also time dependent [44].

Results from randomized, double-blind, phase III clinical trials have demonstrated that ceftobiprole monotherapy is noninferior to ceftriaxone both as monotherapy and in combination with linezolid when treating severe CAP [45].

In 2008, the US Food and Drug Administration (FDA) declined to approve ceftobiprole. However, in 2015, it was designed as an infectious disease product for the treatment of pulmonary and skin infectious by the FDA [46]. In contrast, in 2013, ceftobiprole was approved by the European Medicine Agency (EMA) for the treatment of CAP and hospital-acquired pneumonia, excluding ventilator-associated pneumonia, at a recommended intravenous dose of 500 mg every 8–12 h in adults [47].

Ceftaroline is a fifth-generation extended-spectrum cephalosporin that binds to penicillin binding proteins and prevents bacterial cell wall synthesis. Its antimicrobial activity is directed against gram-positive organisms, including *S. pneumoniae*, *Streptococcus pyogenes*, *S. aureus* (including MRSA, vancomycin-resistant *S. aureus*, and hetero-resistant vancomycin intermediate *S. aureus*), as well as many common gram-negative organisms, such as *Haemophilus influenzae* and *Moraxella catarrhalis*. Phase III clinical trials (FOCUS 1 and 2) have found that ceftaroline is noninferior to ceftriaxone for the treatment of CAP, with cure rates exceeding 82% [48,49]. Ceftaroline is usually well-tolerated, and in clinical trials, only 3% of subjects discontinued therapy due to adverse effects. The most common adverse effects were rash, diarrhea, headache, hypokalemia, insomnia, and phlebitis. The recommended adult dosage is 600 mg/12 h intravenously over 1 h for 5–7 days.

Cedftaroline was approved in 2010 for the treatment of acute bacterial skin, skin structure infections (ABSSSIs), and community-acquired bacterial pneumonia by the FDA [50]. In 2012, it was approved by the EMA for the treatment of complicated skin and soft tissue infections and community-acquired pneumonia [51].

Omadacycline is a semi-synthetic aminomethylcycline derived from minocycline. It has shown a broad spectrum of antimicrobial activity against aerobic and anaerobic gram-positive bacteria (*S. pneumoniae*, *S. aureus*, MRSA), gram-negative bacteria (*Haemophilus influenzae*, *Klebsiella pneumoniae*), and atypical bacteria (*Chlamydophila pneumoniae*, *Legionella pneumophila*, and *Mycoplasma pneumoniae*). Moreover, it has demonstrated activity against MDR bacteria. Omadacycline binds to the 30S ribosomal subunit and blocks bacteria protein synthesis in bacteria [52]

In a phase III clinical trial (OPTIC study) [53], omadacycline was noninferior to moxifloxacin for the treatment of bacterial CAP. Patients were randomized to IV omadacycline 100 mg every 12 h for two doses followed by 100 mg/day or moxifloxacin 400 mg/day for 3 days, both intravenously, with the option to switch to oral therapy or continue for a total of 7–14 days. In the intention-to-treat population, omadacycline performed similarly to moxifloxacin at the early clinical response evaluation (81.1% and 82.7%, respectively). At the posttreatment evaluation, the efficacies of omadacycline versus moxifloxacin were similar in both the intention-to-treat (87.6% vs. 85.1%) and the clinically evaluable populations (92.9% and 90.4%, respectively). The recommended dosages for omadacycline in bacterial CAP are as follows:

- **Loading dose:** 200 mg by intravenous infusion over 60 min on day 1 or 100 mg by intravenous infusion over 30 min twice on day 1.
- **Maintenance dose:** 100 mg by intravenous infusion over 30 min once daily or 300 mg orally once daily.
- **Duration:** 7 to 14 days.

In 2018, omadacycline was approved by the EMA and FDA for community-acquired bacterial pneumonia, acute bacterial skin, and skin structure infections [52,54].

Lefamulin is a novel semisynthetic pleuromutilin that inhibits bacterial protein synthesis. It binds to the peptidyl transferase center of the 50s bacterial ribosome, preventing the binding of transfer RNA for peptide transfer. Lefamulin expresses antimicrobial activity against gram-positive (*S. pneumoniae*, *H. influenza*) and intracellular pathogens (*Mycoplasma pneumoniae*, *Legionella pneumophila*, and *Chlamydophila pneumoniae*) associated with CAP but also has activity against MRSA and *vancomycin-resistant Enterococci*.

In a phase III clinical trial (LEAP 1) [55], the efficacy and safety of lefamulin (intravenous and oral) were compared with those of moxifloxacin (with or without linezolid). The study included 551 adult patients with bacterial CAP, of whom 276 patients were randomized to receive lefamulin 150 mg every 12 h and 275 were randomized to receive moxifloxacin 400 mg every 24 h (with or without linezolid depending on the clinical suspicion of infection by MRSA). Lefamulin was noninferior to moxifloxacin for the primary the Food and Drug Administration (FDA) efficacy outcome of early clinical response (87.3% for lefamulin vs. 90.2% for moxifloxacin +/− linezolid; a 2.9% difference, 95%CI −8.5 to 2.8). The new antibiotic also met the European Medicines Agency non-inferiority endpoint of investigator assessment of clinical response (IACR) at a test-of-cure visit 5–10 days after therapy (86.9% for lefamulin vs. 89.4% for moxifloxacin +/−linezolid; 2.5% difference, 95% CI −8.4 to 3.4).

In a second phase III clinical trial (LEAP 2) [56], the efficacy and safety of lefamulin (5 days oral) were compared with those for moxifloxacin (7 days oral) among 738 adults with moderately severe bacterial CAP. Lefamulin met the FDA primary endpoint of non-inferiority (10.0% margin) for an early clinical response at 72–120 h following therapy in the intention-to-treat population. Early clinical response was 90.8% after 5 days of treatment with lefamulin and 90.8% after 7 days of treatment with moxifloxacin (treatment difference, 0.1; 95% CI −4.4 to 4.5). Lefamulin also met the EMA primary endpoint of non-inferiority (10.0% margin) based on an IACR of 5–10 days following drug dosing in the modified intention-to-treat and clinically evaluable at test-of-cure populations. The IACR rates for the modified intention-to-treat population were 87.5% for lefamulin and 89.1% for moxifloxacin (treatment difference −1.6; 95% CI −6.3 to 3.1]), whereas for the clinically evaluable at test-of-cure population, they were 89.7% for lefamulin and 93.6% for moxifloxacin (treatment difference −3.9; 95% CI −8.2 to 0.5]). Lefamulin is currently undergoing FDA and EMA review for the treatment of CAP, both in intravenous and oral formulations.

## 7. Conclusions

It is crucial that we identify patients with severe CAP at risk of being infected with PES pathogens. Specific risk factors, the local ecology, and resistance patterns should always be considered when determining the most appropriate empirical antibiotic therapy. Our recommendation is to follow the current international guidelines for empiric therapy in severe CAP and to use the PES score to categorize patients at risk of infection with PES pathogens, reserving the coverage for PES pathogens for patients at high risk (i.e., PES score ≥5 points). Of course, clinicians will need to become aware of the pharmacological characteristics and microbial activities of the new antibiotics approved for the management of CAP, especially their broad spectrum of coverage.

## Figures and Tables

**Table 1:** The ATS/IDSA severity criteria for community-acquired pneumonia. Adapted from reference [1].

Major Criteria
Invasive mechanical ventilation
Septic shock
**Minor Criteria**
Blood urea nitrogen level ≥20 mg/dL (7.14 mmol/L)
Confusion/disorientation
Hypotension requiring aggressive fluid resuscitation
Hypothermia (core temperature <96.8°F (36 °C))
Leukopenia (white blood cell count <4000 cells/mm^3^ (4.00 × 10^9^/L))
Multilobar infiltrates
PaO_2_/FiO_2_ ratio ≤250
Respiratory rate ≥30 breaths/minute
Thrombocytopenia (platelets <100 × 10^3^ cells/mm^3^ (100 × 10^9^/L))

ATS/IDSA, American Thoracic Society/Infectious Disease Society of America; FiO_2_, fraction of inspired oxygen; PaO_2_, partial arterial oxygen pressure.

**Table 2:** Definitions of the various categories of drug resistance. Adapted from Reference [29].

Category	Definition
Multidrug resistance (MDR)	Non-susceptibility to at least one agent in three or more antimicrobial categories
Extensively drug resistance (XDR)	Non-susceptibility to at least one agent in all but two or fewer antimicrobial categories
Pan drug resistance (PDR)	Non-susceptibility to all agents in all antimicrobial categories

**Table 3:** PES score. Adapted from Reference [29].

Score to PES Pathogen	Points
Age > 65	1 point
Male	2 point
Previous antibiotic use	2 point
Chronic respiratory disorder	2 point
Chronic renal disease	2 point
**At Emergency**	
Consciousness impairment or aspiration evidence	2 point
Fever or shivers	−1 point

Low risk MDR score: ≤1; Medium risk MDR score: 2-4; High risk MDR score: ≥5. PES (*Pseudomonas aeruginosa*, *Enterobacteriaceae* extended spectrum β-lactamase-positive, and methicillin-resistant *Staphylococcus aureus*).
