# Addendum: Cillóniz, C.; Dominedò, C.; Nicolini, A.; Torres, A. PES Pathogens in Severe Community-Acquired Pneumonia. *Microorganisms* 2019, *7*, 49

**DOI:** 10.3390/microorganisms7060168

**Published:** 2019-06-06

**Authors:** Catia Cillóniz, Cristina Dominedò, Antonello Nicolini, Antoni Torres

**Affiliations:** 1 Department of Pneumology, Hospital Clinic of Barcelona—Institut d’Investigacions Biomèdiques August Pi i Sunyer (IDIBAPS), University of Barcelona (UB)—SGR 911—Ciber de Enfermedades Respiratorias (Ciberes), 08036 Barcelona, Spain; catiacilloniz@yahoo.com; 2 Department of Anesthesiology and Intensive Care Medicine, Fondazione Policlinico Universitario A. Gemelli, Università Cattolica del Sacro Cuore, 00168 Rome, Italy; c.dominedo1@alice.it; 3 Respiratory Diseases Unit, Hospital of Sestri Levante, 16039 Sestri Levante, Italy; antonellonicolini@gmail.com

In the article recently published in *Microorganisms* [1], there was a mistake in the text about PES score and in Table 3 (“PES score”). The authors apologize for this error.

The correct text and Table 3 are provided below: 

In 2015, Prina et al. [31] proposed the PES score, based on the three most frequent pathogens outside the core microorganisms of CAP (e.g., *P. aeruginosa*, extended-spectrum β-lactamase-positive *Enterobacteriaceae*, and methicillin-resistant *S. aureus*). The following elements were included: 0 point for age <40 years; 1 point each for age 40–65 years and male sex; 2 points each for age >65 years, previous antibiotic use, chronic respiratory disorder, and impaired consciousness; 3 points for chronic renal failure; and minus 1 point if fever was present initially. The thresholds ≤1 point, 2–4 points, and ≥ 5 points indicated low, medium, and high risk of PES pathogens, respectively (Table 3).

## Figures and Tables

**Table 3:** PES score. Adapted from Reference [31].

Score to PES Pathogen	Points
Age <40 years	0
Age 40–65 years	1
Age >65 years	2
Male sex	1
Previous antibiotic use	2
Chronic respiratory disorder	2
Chronic renal failure	3
**At Emergency**	
Consciousness impairment or aspiration evidence	2
Fever or shivers	−1

PES (*Pseudomonas aeruginosa*, extended-spectrum β-lactamase-positive *Enterobacteriaceae*, and methicillin-resistant *Staphylococcus aureus*). Low risk, MDR score: ≤1; medium risk, MDR score: 2–4; high risk, MDR score: ≥5.
